# In-hospital Outcomes and Early Hemodynamic Management According to Echocardiography Use in Hypotensive Preterm Infants: A National Propensity-Matched Cohort Study

**DOI:** 10.3389/fcvm.2022.852666

**Published:** 2022-07-14

**Authors:** Roberto Raschetti, Héloïse Torchin, Laetitia Marchand-Martin, Géraldine Gascoin, Gilles Cambonie, Olivier Brissaud, Jean-Christophe Rozé, Laurent Storme, Pierre-Yves Ancel, Armand Mekontso-Dessap, Xavier Durrmeyer

**Affiliations:** ^1^Neonatal Intensive Care Unit, CHI Créteil, Créteil, France; ^2^Université Paris Cité, CRESS, INSERM, INRA, Paris, France; ^3^Assistance Publique-Hôpitaux de Paris, Department of Neonatal Medicine, Maternité Cochin-Port Royal, Paris, France; ^4^Department of Neonatal Medicine, Toulouse University Hospital, Toulouse, France; ^5^Department of Neonatal Medicine, Montpellier University Hospital, Montpellier, France; ^6^Department of Pediatric and Neonatal Intensive Care, Hôpital Pellegrin-Enfants, CHU Pellegrin, Université Bordeaux II, Bordeaux, France; ^7^Department of Neonatal Medicine, Nantes University Hospital, Nantes, France; ^8^Department of Neonatal Medicine, Lille University Hospital, Lille, France; ^9^Assistance Publique-Hôpitaux de Paris, Medical Intensive Care Unit, Centre Hospitalier Universitaire Henri Mondor, Créteil, France; ^10^Université Paris Est Créteil, Faculté de Médecine de Créteil, IMRB, GRC CARMAS, Créteil, France

**Keywords:** hypotension, preterm infants, neonatologist-performed echocardiography, antihypotensive treatments, hemodynamic

## Abstract

**Background:**

Hypotension is a common condition during the first postnatal days of very preterm infants and has been associated with an increased risk of adverse outcomes but its management remains controversial. There is a consensus to promote the use of neonatologist-performed echocardiography (NPE) in hypotensive very preterm infants, although no clinical trial ever assessed this practice.

**Methods:**

We conducted a retrospective analysis of prospectively collected data from the French national EPIPAGE-2 cohort to evaluate the association of NPE with survival, severe morbidity, and therapeutic management in very preterm infants with early hypotension. Reasons for administering antihypotensive treatments were also analyzed. We included infants born before 30 weeks of gestation with hypotension within 72 h of birth. Infants managed with (NPE group) or without (no-NPE group) NPE use were compared after matching on gestational age and a propensity score, reflecting each patient's probability of having an NPE based on his/her baseline covariates. This matching procedure intended to control for the indication bias of NPE.

**Results:**

Among 966 eligible infants, 809 were included (NPE group, *n* = 320; no-NPE group, *n* = 489), and 229 from each group could be matched. The NPE group did not differ significantly from the no-NPE group for survival (OR 1.01, 95% CI 0.64 to 1.60; *p* = 0.95) or survival without severe morbidity at discharge (OR 0.92, 95% CI 0.63 to 1.34; *p* = 0.66), but received more antihypotensive treatments [144/229 (62.9%) vs. 99/229 (43.0%), *p* < 0.001]. Isolated hypotension was the main reason for treatment in both groups. Among treated infants, volume expansion was administered at equal rates to the NPE and no-NPE groups [118/144 (82.1%) vs. 79/99 (80.1%), *p* = 0.67], but the NPE group received inotropic drugs more often [77/144 (53.7%) vs. 37/99 (37.8%), *p* = 0.023].

**Conclusion:**

NPE use in hypotensive preterm infants was not associated with in-hospital outcomes and had little influence on the nature of and reasons for antihypotensive treatments. These results suggest the need to optimize NPE use.

## Background

Low arterial blood pressure, often referred to as hypotension, is a common condition during the first postnatal days of very premature infants and has been associated with an increased risk of death or other adverse outcomes such as bronchopulmonary dysplasia or intraventricular hemorrhage (1).

Although hypotension management remains controversial (2–5), there is a consensus to promote multimodal hemodynamic assessment in premature infants, especially with the use of neonatologist-performed echocardiography (NPE) (6). By providing a more comprehensive assessment of the pathophysiological mechanisms leading to low systemic perfusion than blood pressure measurement alone, NPE could theoretically help clinicians decide whether or not to start treatment, and if yes, to choose the most appropriate treatment strategy (7). NPE has been adopted in many NICUs in recent decades (8) and has been shown to influence therapeutic decisions in studies with historical control groups (9, 10), but its impact on clinical outcomes has been assessed only once, when it was used to systematically screen for patent ductus arteriosus (PDA) among preterm infants born before 30 weeks of gestation (11).

The French population-based EPIPAGE-2 (EPIdémiologie des Petits Ages GEstationnels) prospective cohort study recruited premature births in 2011 (12) and collected the use of NPE to assess hemodynamics in the first 72 h after birth. These real-life data offer a unique opportunity to evaluate the association of NPE use with therapeutic management and outcomes in hypotensive preterm infants in a situation where randomization of this imaging practice at the level of a unit seems difficult.

We thus aimed to compare survival, survival without significant morbidity at discharge, and early hemodynamic therapeutic management in preterm infants born before 30 weeks of gestational age who had an episode of hypotension and did or did not have at least one NPE during their first 72 postnatal hours, after adjustment for confounding by indication. We hypothesized that babies in whom NPE was used would receive a specific hemodynamic management and would thus have better outcomes at discharge.

## Methods

### Study Design and Data Source

This was a retrospective analysis of prospectively collected data from the EPIPAGE-2 cohort, a French birth cohort intended to describe perinatal management and short- and long-term outcomes of preterm infants (12, 13). Briefly, from March 2011 through December 2011, all maternity units in France included premature births during an 8-month period for births occurring at 24–26 weeks and a 6-month period for births at 27–29 weeks. Data were collected by neonatal and obstetric teams from medical records in specific standardized questionnaires and verified by the local pediatric study coordinator (12).

### Participants

Neonates were eligible for this study if they were born between 24^+0^ and 29^+6^ weeks of gestation, were admitted to a participating NICU, and had at least one episode of hypotension defined as a minimum mean arterial blood pressure value (minMAP) in mm Hg lower than GA in weeks (minMAP < GA) before 72 h after birth (1, 3, 4). Blood pressure could be measured invasively or non-invasively, and the frequency of blood pressure measurements was not collected.

Exclusion criteria were treatment limitation or withdrawal within 72 h after birth, lethal congenital malformations, and missing data for NPE, minMAP, or hemodynamic treatment in the first 72 h after birth.

### Exposure

Exposure was defined as the use of NPE to assess hemodynamic status within the first 72 h after birth. If NPE was performed, neither the number of scans nor the timing of NPE, i.e., whether it preceded or followed a therapeutic decision, was collected. This item in the questionnaire was distinct from that about the systematic echocardiographic screening of PDA. The “NPE” group included infants who received at least one NPE for hemodynamic assessment within 72 h after birth, and the “no-NPE” group infants who did not receive NPE in that period.

### Outcomes

Primary outcomes were survival to hospital discharge and survival to discharge without severe morbidity, defined as any of the following: severe bronchopulmonary dysplasia, defined as administration of oxygen for at least 28 days plus need for 30% or more oxygen and/or mechanical ventilatory support or continuous positive airway pressure at 36 weeks' postmenstrual age; stage II and III necrotizing enterocolitis according to Bell's staging; severe retinopathy of prematurity (ROP), defined as stage 3 or more and/or requiring treatment; any of the following severe cerebral abnormalities on cranial ultrasonography: grade III intraventricular hemorrhage according to Volpe's classification; intraparenchymal hemorrhage, defined as a large unilateral parenchymal hyperdensity or a large unilateral porencephalic cyst, or cystic periventricular leukomalacia, defined as periventricular white matter echolucencies. We considered the most severe brain lesion observed among all brain ultrasounds performed until discharge or death.

Secondary outcomes included each of the previously mentioned severe morbidities.

For exploratory analyses, we examined the use of antihypotensive treatments in the first 3 days after birth, their type, and the main reason declared by the attending physician for using such therapy (see definitions in the Supplementary Material).

### Statistical Analysis

#### Primary Analysis

To control for the non-random exposure to NPE, we constructed a multivariable logistic regression model to estimate each patient's probability (i.e., propensity score) of having an NPE based on his/her baseline covariates. This model included two types of variables, associated with exposure and/or outcomes: maternal and pregnancy-related characteristics, and neonatal characteristics (see details in the Supplementary Material).

To assess the average treatment effect related to NPE use in the treated infants (ATT), we used a 1:1 greedy matching algorithm without replacement to match exposed and non-exposed infants for gestational age and propensity score within a caliper of 0.2 standard deviations of the logit of the propensity score (14). Standardized differences were examined to assess balance in the observed baseline covariates between exposed and non-exposed groups, with a threshold of 10%, above which the imbalance between groups was unacceptable (15).

In the unmatched cohort, all percentages were weighted to take differences in the recruitment periods into account for infants born at 24^+0^-26^+6^ weeks and at 27^+0^-29^+6^ weeks.

Outcomes were compared between the NPE and no-NPE groups with odds ratios (ORs) calculated with logistic regression fit by generalized estimating equations (GEE) to account for paired data. The Chi-square test was used to compare the NPE and no-NPE groups for the frequency and nature of antihypotensive treatments and the reason for their use. Median values of volume expansion between the NPE and no-NPE groups were compared with Wilcoxon's Rank-Sum test.

Missing baseline and outcome variables were handled with multiple imputations by chained equations that used the other available variables. We generated 50 independent imputed datasets with 30 iterations each, pooled according to Rubin's rule (16). All tests were 2-sided, and *p*-values < 0.05 were considered significant. All analyses were performed with R (version 3.6.1) and SAS (version 9.4) software.

#### Sensitivity Analyses

Two sensitivity analyses were performed using inverse probability of treatment weighting (IPTW) and a GEE regression analysis (see details in the Supplementary Material).

#### Subgroup Analyses

Two *post-hoc* subgroup analyses were performed according to the severity of hypotension (minMAP ≤ or > GA-5) and exposure to antihypotensive treatments (treated or untreated). A new propensity score was calculated in each subgroup, and patients were matched as in the main analysis.

## Results

**Figure 1:**
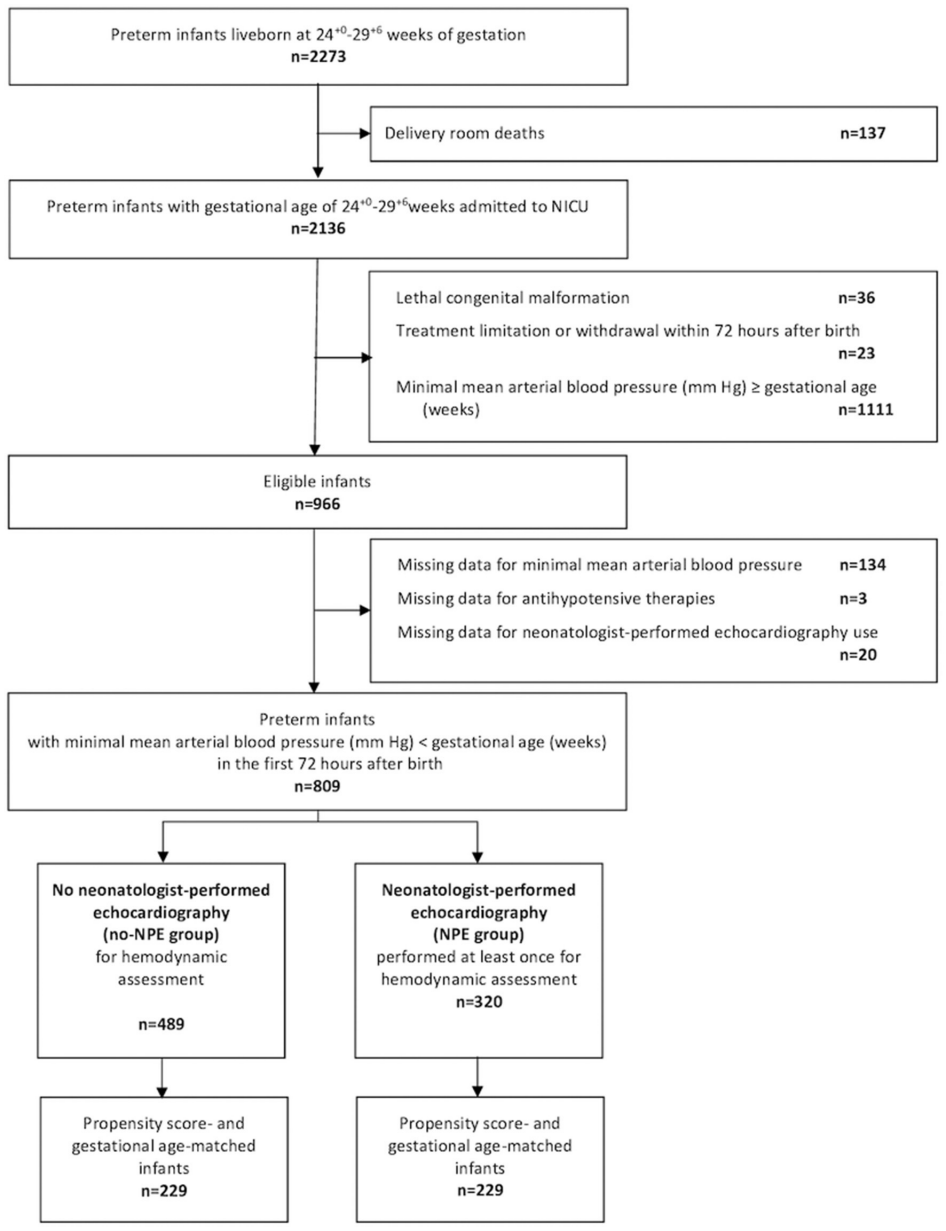
Population flow chart. NICU, neonatal intensive care unit.

**Figure 2:**
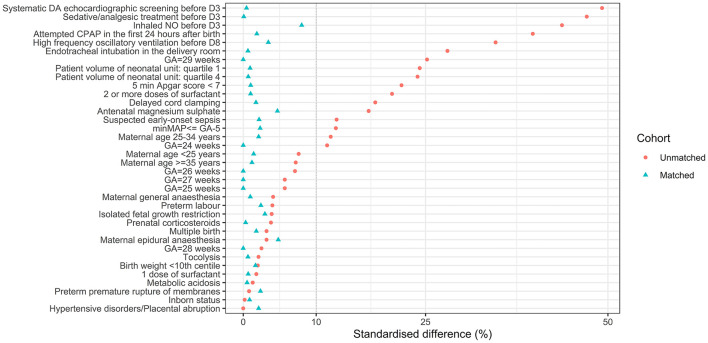
Standardized difference (SDs) in the unmatched and matched cohort, after multiple imputation. SDs are represented on the X-axis. Each covariate included in the propensity score is on the Y-axis. Red dots represent values for the initial sample after weighting to take into account the difference in recruitment periods between gestational age groups and after imputation. Blue triangles illustrate values for the matched cohort. The dotted line represents the 10% standardized difference, which is usually considered as the threshold above which balance in a propensity score is unacceptable. D3, day three after birth; D8, day eight after birth; GA, gestational age; NO, nitric oxide; CPAP, continuous positive airway pressure; minMAP, minimal mean arterial blood pressure.

Among the 2,136 premature infants born before 30 weeks of gestation in the EPIPAGE-2 cohort and admitted to the NICU, 966 were eligible and 809 were included in the study (Figure 1). Baseline characteristics of the unmatched and matched cohorts are summarized in Table 1. Of the 320 infants in the NPE group and 489 in the no-NPE group, 229 from each group could be matched for gestational age and propensity score (Figure 1). In the unmatched cohort, infants in the NPE group had more frequent markers of severity but matched pairs had standardized differences below 10% for all variables included in the propensity score (Figure 2). The overlap of propensity scores in the NPE and no-NPE groups was limited for extreme values meaning that most infants with a high probability of having NPE actually did and that most infants with a low probability of having it did not (online Supplementary Figure 1).

**Table 1:** Baseline characteristics according to neonatologist-performed echocardiography use.

	**Unmatched cohort** ^**a**^				**Matched cohort** ^**b**^	
			**After multiple imputation**			
	**no NPE (*n* = 489)**	**NPE (*n* = 320)**	**no NPE**	**NPE**	**no NPE (*n* = 229)**	**NPE (*n* = 229)**
	***n* (%)**	***n* (%)**	**%**	**%**	***n* (%)**	***n* (%)**
**Maternal characteristics at birth**						
**Maternal age**						
<25 years	88 (17.8)	49 (15.0)	17.8	15.0	39 (17.0)	38 (16.5)
25–34 years	284 (58.0)	205 (63.9)	58.0	63.9	142 (61.8)	144 (62.9)
>35 years	117 (24.2)	66 (21.1)	24.2	21.1	48 (21.2)	47 (20.7)
**Prenatal corticosteroids**	387 (81.6)	264 (82.7)	81.0	82.5	186 (81.1)	185 (81.0)
Missing	11	2	NA	NA	NA	NA
**Tocolysis**	286 (57.9)	182 (56.6)	57.8	56.8	130 (56.5)	129 (56.2)
Missing	1	2	NA	NA	NA	NA
**Antenatal Mg sulfate**	33 (7.2)	38 (12.1)	7.2	12.3	22 (9.6)	25 (11.0)
Missing	4	8	NA	NA	NA	NA
**Cause of preterm birth**						
Preterm labor	222 (44.7)	140 (42.7)	44.7	42.7	105 (46.0)	102 (44.7)
Preterm premature rupture of membranes	121 (24.3)	80 (24.6)	24.3	24.6	49 (21.5)	52 (22.5)
Hypertensive disorder and/or placental abruption	97 (20.6)	62 (20.6)	20.6	20.6	47 (20.7)	49 (21.6)
Isolated fetal growth restriction	18 (3.9)	14 (4.7)	3.9	4.7	12 (5.2)	11 (4.6)
Other	31 (6.5)	24 (7.3)	6.5	7.3	15 (6.6)	15 (6.5)
**Cesarean section**	292 (61.4)	202 (65.2)	61.2	65.3	149 (64.8)	147 (64.1)
Missing	2	1	NA	NA	NA	NA
**Maternal anesthesia**						
General	83 (16.9)	56 (18.3)	16.7	18.3	40 (17.5)	41 (17.9)
Epidural	320 (67.3)	219 (68.9)	67.4	68.9	159 (69.4)	154 (67.2)
No anesthesia	80 (15.8)	42 (12.7)	15.9	12.8	30 (13.0)	34 (14.9)
Missing	6	3	NA	NA	NA	NA
**Multiple birth**	158 (32.1)	110 (33.6)	32.1	33.6	79 (34.7)	77 (33.8)
**Neonatal characteristics at birth**						
**Gestational age**						
24 weeks	20 (3.3)	22 (5.7)	3.3	5.7	8 (3.6)	8 (3.6)
25 weeks	67 (11.2)	50 (13.1)	11.2	13.1	35 (15.4)	35 (15.4)
26 weeks	89 (14.9)	67 (17.5)	14.9	17.5	48 (21.0)	48 (21.0)
27 weeks	91 (20.5)	65 (22.9)	20.5	22.9	55 (24.0)	55 (24.0)
28 weeks	103 (23.2)	69 (24.3)	23.2	24.3	48 (21.0)	48 (21.0)
29 weeks	119 (26.8)	47 (16.5)	26.8	16.5	35 (15.1)	35 (15.1)
**Male sex**	254 (51.3)	173 (54.5)	51.3	54.5	122 (53.3)	122 (53.2)
**Birth weight** **<** **10**^**th**^ **centile**^**c**^	140 (29.6)	96 (30.5)	29.6	30.5	69 (30.2)	71 (30.9)
**Delayed cord clamping**	4 (0.9)	14 (4.5)	1.4	4.5	6 (2.6)	7 (2.9)
Missing	26	7	NA	NA	NA	NA
**5-min Apgar score** **<** **7**	98 (21.7)	96 (32.2)	22.6	32.2	65 (28.4)	66 (28.8)
Missing	45	25	NA	NA	NA	NA
**Metabolic acidosis** ^**d**^	125 (28.4)	83 (28.8)	28.5	29.1	67 (29.4)	67 (29.1)
Missing	54	37	NA	NA	NA	NA
**Endotracheal intubation in the delivery room**	398 (80.3)	290 (90.2)	80.3	90.2	204 (89.3)	204 (89.0)
Missing	2	0	NA	NA	NA	NA
**Surfactant**						
No	70 (15.7)	14 (4.9)	15.7	5.2	15 (6.3)	15 (6.5)
1 dose	267 (55.2)	171 (56.5)	55.2	56.1	130 (56.7)	131 (57.0)
>2 doses	147 (29.1)	119 (38.6)	29.1	38.7	85 (37.0)	84 (36.5)
Missing	5	16	NA	NA	NA	NA
**Attempted CPAP in the first 24 hours after birth**	232 (51.5)	92 (32.5)	50.1	31.0	83 (36.1)	81 (35.2)
Missing	22	30	NA	NA	NA	NA
**High-frequency oscillatory ventilation before D8**	90 (20.5)	101 (36.3)	20.6	36.0	66 (28.8)	70 (30.4)
Missing	63	42	NA	NA	NA	NA
**Inhaled NO before D3**	17 (3.5)	52 (16.3)	3.6	16.4	16 (7.0)	21 (9.4)
Missing	7	6	NA	NA	NA	NA
**Suspected early-onset sepsis**	118 (24.5)	95 (30.4)	24.7	30.4	63 (27.6)	66 (28.6)
Missing	13	12				
**Sedative and/or analgesic treatment before D3**	187 (37.5)	193 (60.4)	37.5	60.4	118 (51.4)	118 (51.4)
Missing	1	0	NA	NA	NA	NA
**Systematic DA echocardiographic screening before D3**	187 (38.6)	195 (63.1)	39.1	63.0	131 (57.3)	132 (57.5)
Missing	13	12	NA	NA	NA	NA
**MinMAP** **≤GA-5**	183 (38.1)	139 (44.3)	38.1	44.3	101 (44.1)	98 (43.0)
**Inborn status**	429 (88.1)	283 (88.2)	88.1	88.2	200 (87.3)	199 (87.0)
**Patient volume of neonatal unit** ^**e**^						
<30	123 (25.5)	52 (15.8)	25.5	15.8	43 (18.8)	42 (18.4)
[30–45[	145 (29.3)	89 (28.3)	29.3	28.3	73 (31.8)	71 (30.9)
[45–60[	82 (17.2)	120 (37.9)	17.2	37.9	65 (28.4)	67 (29.4)
≥60	139 (28.0)	59 (18.0)	28.0	18.0	48 (21.0)	49 (21.3)

^a^*Percentages were weighted to take into account the differences in survey design by gestational age group. ^b^Matching by gestational age in weeks and propensity score after multiple imputation. Mean numbers and percentages among the 50 imputed datasets. ^c^Based on French intrauterine growth curves (17). ^d^Defined as a base excess < -7 in the first 12 h of life. ^e^Defined by the number of preterm infants <30 weeks of gestation included in the EPIPAGE 2-study in the unit, divided into quartiles. NPE, neonatologist-performed echocardiography; CPAP, continuous positive airway pressure; NO, nitric oxide; D8, day eight after birth; D3, day three after birth; DA, ductus arteriosus; minMAP, minimum mean arterial blood pressure; GA, gestational age*.

In the unmatched cohort, 213 (66.9%) of 320 infants from the NPE group received antihypotensive therapies vs. 169 (34.1%) of 489 in the no-NPE group (*p* < 0.001, Table 2). Survival at discharge did not differ significantly between the NPE and no-NPE groups [244/320 (77.5%) vs. 396/489 (82.4%), respectively; *p* = 0.08; Table 3] but survival without severe morbidity was lower in the NPE than in the no-NPE group [156/320 (50.7%) vs. 281/489 (59.8%), respectively; *p* = 0.012; Table 3].

**Table 2:** Antihypotensive treatments, PDA treatments, and main reason for treatment in infants in NPE and no-NPE groups.

	**Unmatched cohort** ^**a**^			**Matched cohort** ^**b**^		
	**no NPE (*n* = 489)**	**NPE (*n* = 320)**	***p*-value^**c**^**	**no NPE (*n* = 229)**	**NPE (*n* = 229)**	***p*-value^**c**^**
	***n* (%)**	***n* (%)**		***n* (%)**	***n* (%)**	
**Antihypotensive treatment before D3**	169 (34.1)	213 (66.9)	<0.001	99 (43.2)	144 (62.9)	<0.001
**Among treated infants**	*n* = 169	*n* = 213	–	*n* = 99	*n* = 144	–
**Volume expansion**	132 (78.1)	174 (82.2)	0.32	79 (80.5)	118 (82.1)	0.67
Volume in ml/kg, median (IQR)	20 (10–30)	20 (17–40)	0.003	20 (13–30)	20 (16–40)	0.013
Missing	2	15		1	8	
**Inotropic drugs**	59 (33.9)	118 (54.8)	<0.001	37 (37.8)	77 (53.7)	0.023
if any	*n* = 59	*n* = 118		*n* = 37	*n* = 77	
Dopamine	51 (85.9)	89 (76.1)	0.14	31 (84.5)	61 (78.6)	0.49
Dobutamine	12 (20.9)	31 (26.1)	0.46	9 (23.1)	16 (21.2)	0.69
Norepinephrine	9 (15.5)	22 (18.7)	0.61	7 (18.2)	12 (15.1)	0.61
**Corticosteroids**	56 (33.3)	61 (27.2)	0.20	35 (36.0)	37 (25.3)	0.11
**Treatment combinations**						
Volume expansion only	76 (45.7)	72 (34.8)		42 (41.6)	54 (37.2)	
Inotropic drugs only	14 (7.9)	20 (9.0)		8 (8.6)	14 (9.8)	
Corticosteroids only	17 (10.6)	13 (6.0)		8 (8.5)	7 (5.0)	
Volume expansion + inotropic drugs	23 (13.2)	60 (29.0)	0.001	14 (13.9)	40 (27.6)	0.07
Volume expansion + corticosteroids	17 (9.9)	10 (4.4)		12 (12.2)	6 (4.0)	
Inotropic drugs + corticosteroids	6 (3.5)	6 (2.8)		2 (2.4)	4 (3.1)	
Volume expansion + inotropic drugs + corticosteroids	16 (9.4)	32 (14.0)		13 (12.9)	19 (13.2)	
**Declared indication for treatment**						
Isolated hypotension	86 (59.0)	66 (37.5)		48 (55.8)	49 (41.6)	
Hypotension associated with clinical signs of hypoperfusion	60 (40.4)	53 (30.2)		37 (43.0)	38 (32.0)	
Echographic findings and clinical criteria	0 (0.0)	57 (32.3)	<0.001	0 (0.0)	31 (26.5)	<0.001
Echographic findings alone	0 (0.0)	0 (0.0)		0 (0.0)	0 (0.0)	
Others (NIRS, lactate, ...)	1 (0.6)	0 (0.0)		1 (1.2)	0 (0.0)	
Missing	24	37	–	12	26	–
**PDA treatment before D3**						
No treatment	295 (63.7)	118 (38.7)		127 (55.6)	89 (39.0)	
Ibuprofen before D3	65 (12.8)	111 (35.1)	<0.001	43 (18.7)	78 (33.9)	0.001
Ibuprofen or surgery at D3 or after	120 (23.6)	82 (26.3)		59 (25.7)	62 (27.1)	
Missing	9	9	–	0	0	–

^a^*Percentages and p values were weighted to take into account the differences in survey design by gestational age group. ^b^Matching by gestational age in weeks and propensity score after multiple imputation. Mean numbers and percentages among the 50 imputed datasets. ^c^Chi-square test for categorical variables and the Wilcoxon Rank-Sum test for continuous variables. NPE, neonatologist-performed echocardiography; D3, day 3 after birth; PDA, patent ductus arteriosus; NIRS, near infrared spectroscopy*.

**Table 3:** Primary and secondary outcomes according to use of neonatologist-performed echocardiography in the unmatched and matched cohorts after multiple imputation.

	**Unmatched cohort** ^**a**^				**Matched cohort** ^**b**^			
	**no NPE (*n* = 489)**	**NPE (*n* = 320)**	**OR (95%CI)**	***p*-value**	**no NPE (*n* = 229)**	**NPE (*n* = 229)**	**OR (95%CI)**	***p*-value**
	***n* (%)**	***n* (%)**	**NPE vs. no NPE**		***n* (%)**	***n* (%)**	**NPE vs. no NPE**	
**Survival at discharge**	396 (82.4)	244 (77.5)	0.74 (0.52; 1.04)	0.08	178 (77.8)	179 (78.1)	1.01 (0.64; 1.60)	0.95
**Survival at discharge without severe neonatal morbidity** ^**c**^	281 (59.8)	156 (50.7)	0.69 (0.52; 0.92)	0.012	123 (53.5)	118 (51.4)	0.92 (0.63; 1.34)	0.66
**Secondary outcomes**								
Severe cerebral abnormalities	78 (15.2)	63 (19.1)	1.32 (0.91; 1.92)	0.15	42 (18.3)	42 (18.3)	1.00 (0.60; 1.66)	0.99
Necrotizing enterocolitis	19 (4.0)	13 (4.4)	1.11 (0.54; 2.28)	0.78	8 (3.3)	9 (3.8)	1.17 (0.37; 3.67)	0.79
Severe bronchopulmonary dysplasia^d^	64 (15.0)	41 (17.1)	1.26 (0.81; 1.96)	0.31	32 (18.6)	30 (18.2)	0.98 (0.54; 1.76)	0.93
Severe retinopathy of prematurity^d^	5 (1.1)	11 (4.0)	3.73 (1.27; 10.95)	0.017	3 (1.5)	6 (3.3)	2.42 (0.41; 14.13)	0.33

^a^*Weighted to take differences in sampling process by gestational age into account. ^b^Matching by gestational age in weeks and propensity score after multiple imputation. Odds ratios (ORs) were calculated with logistic regression fit by generalized estimating equations (GEE) to take paired data into account. ^c^Severe morbidity was defined as any of: severe bronchopulmonary dysplasia, severe necrotizing enterocolitis, severe retinopathy (stage 3 or treatment needed), or any of the following severe cerebral abnormalities on cranial ultrasonography: intraventricular hemorrhage with ventricular dilatation (Grade III or intraparenchymal hemorrhage, or cystic periventricular leukomalacia. ^d^Among survivors at discharge. NPE, neonatologist-performed echocardiography*.

In the matched cohort, 144 (62.9%) of 229 infants from the NPE group received antihypotensive therapies vs. 99 (43.0%) of 229 infants in the no-NPE group (*p* < 0.001, Table 2). Among infants treated with antihypotensive therapies, the most frequent treatment was volume expansion, administered to 118 (82.1%) of 144 treated infants in the NPE group and to 79 (80.5%) of 99 treated infants in the no-NPE group (*p* = 0.67, Table 2). The median amount of administered volume was significantly higher in the NPE group than in in the no-NPE group [20 (13–30) vs. 20 (16–40) ml/kg, *p* = 0,013; Table 2].

Among infants treated with antihypotensive therapies, the use of inotropic drugs was more frequent in the NPE than the no-NPE group [77/144 (53.7%) vs. 37/99 (37.8%), respectively; *p* = 0.023; Table 2]. The comparison of antihypotensive treatment combinations did not reach a statistically significant difference between the two groups (*p* = 0.07, Table 2).

Reasons for administering antihypotensive treatments differed significantly between the groups (*p* < 0.001), but the most frequently reported reason was isolated hypotension in both the NPE (49/144, 41.6%) and no-NPE (48/99, 56.1%) groups. More infants in the NPE than the no-NPE group received a treatment to close the PDA in their first 3 days after birth [78/229 (33.9%) vs. 43/229 (18.7%), respectively; *p* = 0.001; Table 2].

In the matched cohort, no significant difference between the NPE and no-NPE groups was found for survival (OR 1.01, 95% CI 0.64 to 1.60; *p* = 0.95; Table 3) or survival without severe morbidity at discharge (OR 0.92, 95% CI 0.63 to 1.34; *p* = 0.66; Table 3).

Sensitivity analyses using IPTW and logistic regression provided results similar to those of the main analysis (Supplementary Figure 2).

Subgroup analyses stratified for the severity of hypotension yielded no significant differences between the NPE and no-NPE groups among those with minMAP ≤ or > GA-5 (Supplementary Table 1) or among treated or untreated infants (Supplementary Table 2).

## Discussion

In this real-life nationwide prospective study, NPE was used to assess hemodynamic status in around 40% of preterm infants born before 30 weeks of gestation with hypotension occurring in the first three postnatal days. After adjusting for confounding by indication, we found no association between NPE use and in-hospital survival or survival without severe morbidities. NPE was associated with more frequent use of antihypotensive therapies, but the nature of these therapies was very similar whether NPE was used or not, except for the amount of volume expansion, which was larger in the NPE group. This study included a large sample of premature infants and used robust statistical methods, which contribute to the external validity of the findings.

The use of NPE is variable in NICUs worldwide, ranging from 9% in the United States (18) to 94% in Italy (19). We did not confirm our initial hypothesis that NPE use would be associated with improved outcomes. Actually, many issues must be resolved before evidence-based guidelines predicated on echocardiographic findings can be adopted: effective training must be implemented, relevant and reproducible echocardiographic markers must be identified and widely adopted, and therapeutic interventions based on these markers must be assessed. To date, none of these things has happened: the existing literature on NPE use for guiding hemodynamic management in preterm neonates is currently insufficient (20) and no randomized controlled trial assessing therapeutic options for increasing blood pressure in preterm infants has succeeded in improving important clinical outcomes (21–26). In line with recommendations from adult intensivists (27), standardizing the use of neonatal echocardiography in hemodynamic clinical research should be strongly encouraged.

Contrary to our initial hypothesis, we found little differences between therapeutic interventions among treated infants according to NPE use. Most of the treated hypotensive infants received volume expansion, and the most frequently used inotropic drug was dopamine, as observed in an international survey contemporaneous with the EPIPAGE-2 study (28). Although volume expansion can increase blood pressure, its wide use is not supported by either long-term clinical benefits (22) or pathophysiological mechanisms (5). Similarly, dopamine is effective for increasing blood pressure but does not improve blood flow or clinical outcomes (21). The recently published multicenter, double-blind, placebo-controlled hypotension in preterm infants (HIP) trial failed to establish any definitive conclusion on the effect of dopamine on survival without brain injury due to insufficient recruitment, leaving the relevance of its use in this context unknown (25).

Interestingly, infants in the NPE groups received PDA treatment more frequently in the first three postnatal days than did the no-NPE group, and the latter showed no catch-up PDA treatments afterwards. We speculate that for these infants, NPE might have helped to identify PDA as the cause of hypotension and led to the administration of treatment (29).

Our study has several limitations. First, we had no information on the timing of NPE in relation to the treatment decision. Therefore, we could not distinguish infants who were treated or untreated based on echocardiographic findings from those who underwent NPE once the treatment started. We also had no information on the number of scans and the timeline of any treatment modifications that followed them. Second, the reasons for performing NPE were also missing, so we could speculate that patients in the NPE-group had clinical conditions leading clinicians to perform NPE. This could explain a more frequent use of anti-hypotensive therapies in the NPE group. Third, we had no information on the existence of a local protocol to guide the assessment and management of hypotension in each NICU, which might have influenced the effects of NPE use. Fourth, the study reflects practices from 2011, which might have changed a decade later. Moreover, the questionnaire did not collect the use of epinephrine or milrinone, which were rarely used in France at that time. Fifth, as in any observational study, residual confounding might persist. However, the use of NPE seems impossible to randomize at the level of a single department or hospital, and the feasibility of a cluster-randomized trial is questionable for that would require that some centers already using NPE to agree to stop using it while the other centers would need to follow a proper training program to implement NPE use. Thus, it is likely that the use of NPE can essentially be assessed only through observational studies (30).

## Conclusion

In French NICUs in 2011, NPE was used for the hemodynamic assessment of fewer than half of the very preterm infants with hypotension. NPE use was associated with more frequent use of antihypotensive therapies but had little impact on their type and did not appear to influence survival or survival without severe morbidity at discharge. These results do not argue against the use of NPE in this context but rather underline the gap in our knowledge of how to interpret NPE findings, the need for training and echocardiographic-based clinical research on neonatal hemodynamic management, and the necessity for evidence about the benefits of antihypotensive therapies in this vulnerable population.

## Data Availability Statement

The data analyzed in this study is subject to the following licenses/restrictions: The procedures carried out with the French data privacy authority (CNIL, Commission nationale de l'informatique et des libertés) do not provide for the transmission of the database. Consultation by the editorial board or interested researchers may nevertheless be considered, subject to prior determination of the terms and conditions of such consultation and in respect for compliance with the applicable regulations. Requests to access these datasets should be directed to xavier.durrmeyer@chicreteil.fr.

## Ethics Statement

The studies involving human participants were reviewed and approved by Commission Nationale de l'Informatique et des Libertés (CNIL) n°911009 the consultative committee on the treatment of information on personal health data for research purposes (approval granted November 18, 2010, reference 10.626) the committee for the protection of people participating in Biomedical Research (approval granted March 18, 2011, reference Comité de Protection des Personnes (CPP) SC-2873. Written informed consent from the participants' legal guardian/next of kin was not required to participate in this study in accordance with the national legislation and the institutional requirements.

## Collaboration Group Information

Members of the Hemodynamic EPIPAGE2 Study Group: Gilles Cambonie, MD, PhD (Department of Neonatal Medicine, Montpellier University Hospital, Montpellier, France); Jean-Christophe Rozé, MD, PhD (Department of Neonatal Medicine, Nantes University Hospital, Nantes, France); Pierre-Yves Ancel, MD, PhD (INSERM, U1153,Obstetrical, Perinatal and Pediatric Epidemiology Team, Epidemiology and Biostatistics Sorbonne, Paris, France); Laetitia Marchand-Martin, MS (INSERM, U1153,Obstetrical, Perinatal and Pediatric Epidemiology Team, Epidemiology and Biostatistics Sorbonne, Paris, France); Mélanie Durox, MSc (INSERM, U1153, Obstetrical, Perinatal and Pediatric Epidemiology Team, Epidemiology and Biostatistics Sorbonne, Paris, France); Veronique Gournay, MD, PhD (Pediatric Cardiology Unit, Nantes University Hospital, Nantes, France); Xavier Durrmeyer, MD, PhD (Department of Neonatal Medicine, Intercommunal hospital, Creteil, France); Laurent Storme, MD, PhD (Department of Neonatal Medicine, Lille University Hospital, Lille, France); Raphael Porcher, PhD (INSERM, U1153, METHODS Team, Epidemiology and Statistics Sorbonne Paris Cité Research Center, Paris, France); Patrice Morville, MD (Department of Neonatal Medicine, Reims University Hospital, Reims, France);Olivier Brissaud, MD (Department of Neonatal Medicine, Bordeaux University Hospital, Bordeaux, France); Patrick Truffert, MD, PhD (Department of Neonatal Medicine, Lille University Hospital, Lille, France); Antoine Bouissou, MD (Department of Neonatal Medicine, Tours University Hospital, Tours, France); Isabelle Ligi, MD, PhD (Department of Neonatal Medicine, Marseille University Hospital, Marseille, France); Marie-Odile Marcoux, MD (Department of Neonatal Medicine, Toulouse University Hospital, Toulouse, France); Fabrice Cneude, MD (Department of Neonatal Medicine, Grenoble University Hospital, Grenoble, France); Geraldine Gascoin, MD, PhD (Department of Neonatal Medicine, Angers University Hospital); Gerard Thiriez, MD, PhD (Department of Neonatal Medicine, Besançon University Hospital, Besançon, France); Hugues Patural, MD, PhD (Department of Neonatal Medicine, St Etienne University Hospital, St Etienne, France); Doriane Madeleneau, MD (Department of Neonatal Medicine, Cochin University Hospital, Paris, France); Antoine Burguet, MD, PhD (Department of Neonatal Medicine, Dijon University Hospital, Dijon, France); Patrick Pladys, MD, PhD (Department of Neonatal Medicine, Rennes University Hospital, Rennes, France).

## Author Contributions

GG, GC, J-CR, OB, LS, P-YA, and XD participated in the data collection. LM-M performed all data analysis, and XD validated them. RR, XD, and HT wrote the first draft. All authors initiated and designed the protocol, participated in data interpretation, reviewing of the manuscript, and approved the final manuscript.

## Funding

This study was supported by the French Institute of Public Health Research/Institute of Public Health and its partners the French Health Ministry, the National Institute of Health and Medical Research, the National Institute of Cancer, and the National Solidarity Fund for Autonomy; grant ANR-11-EQPX-0038 from the National Research Agency through the French Equipex Program of Investments in the Future; and the PremUp Foundation. The funders/sponsors had no role in the design and conduct of the study; collection, management, analysis, and interpretation of the data; preparation, review, or approval of the manuscript; or decision to submit the manuscript for publication.

## Conflict of Interest

The authors declare that the research was conducted in the absence of any commercial or financial relationships that could be construed as a potential conflict of interest.

## Publisher's Note

All claims expressed in this article are solely those of the authors and do not necessarily represent those of their affiliated organizations, or those of the publisher, the editors and the reviewers. Any product that may be evaluated in this article, or claim that may be made by its manufacturer, is not guaranteed or endorsed by the publisher.

## Abbreviations

NPE: neonatologist-performed echocardiography
PDA: patent ductus arteriosus
EPIPAGE-2: epidémiologie des petits ages gestationnels
minMAP: minimum mean arterial blood pressure value
ROP: retinopathy of prematurity
ATT: Average Treatment Effect on Treated
ORs: odds ratios
GEE: generalized estimating equations
IPTW: inverse probability of treatment weighting.

## References

[1] Faust K Hartel C Preuss M Rabe H Roll C Emeis M. Short-term outcome of very-low-birthweight infants with arterial hypotension in the first 24 h of life. Arch Dis Child Fetal Neonatal Ed. (2015) 100:F388–92. 10.1136/archdischild-2014-306483 26199082

[2] Batton B Li L Newman NS Das A Watterberg KL Yoder BA. Use of antihypotensive therapies in extremely preterm infants. Pediatrics. (2013) 131:e1865–73. 10.1542/peds.2012-2779 23650301 PMC3666108

[3] Dempsey EM Al Hazzani F Barrington KJ. Permissive hypotension in the extremely low birthweight infant with signs of good perfusion. Arch Dis Child Fetal Neonatal Ed. (2009) 94:F241–4. 10.1136/adc.2007.124263 19174413

[4] Durrmeyer X Marchand-Martin L Porcher R Gascoin G Roze JC Storme L. Abstention or intervention for isolated hypotension in the first 3 days of life in extremely preterm infants: association with short-term outcomes in the EPIPAGE 2 cohort study. Arch Dis Child Fetal Neonatal Ed. (2017) 102:490–6. 10.1136/archdischild-2016-312104 28302697

[5] Schwarz CE Dempsey EM. Management of neonatal hypotension and shock. Semin Fetal Neonatal Med. (2020) 25:101121. 10.1016/j.siny.2020.101121 32473881

[6] de Boode WP Singh Y Gupta S Austin T Bohlin K Dempsey E. Recommendations for neonatologist performed echocardiography in Europe: consensus statement endorsed by European society for paediatric research (ESPR) and European society for neonatology (ESN). Pediatr Res. (2016) 80:465–71. 10.1038/pr.2016.126 27384404 PMC5510288

[7] Singh Y Tissot C Fraga MV Yousef N Cortes RG Lopez J. International evidence-based guidelines on point of care ultrasound (POCUS) for critically ill neonates and children issued by the POCUS working group of the European society of paediatric and neonatal intensive care (ESPNIC). Crit Care. (2020) 24:65. 10.1186/s13054-020-2787-9 32093763 PMC7041196

[8] Roehr CC Te Pas AB Dold SK Breindahl M Blennow M Rudiger M. Investigating the European perspective of neonatal point-of-care echocardiography in the neonatal intensive care unit–a pilot study. Eur J Pediatr. (2013) 172:907–11. 10.1007/s00431-013-1963-1 23440477

[9] El-Khuffash A Herbozo C Jain A Lapointe A McNamara PJ. Targeted neonatal echocardiography (TnECHO) service in a Canadian neonatal intensive care unit: a 4-year experience. J Perinatol. (2013) 33:687–90. 10.1038/jp.2013.42 23619373

[10] Harabor A Soraisham AS. Utility of targeted neonatal echocardiography in the management of neonatal illness. J Ultrasound Med. (2015) 34:1259–63. 10.7863/ultra.34.7.1259 26112629

[11] Roze JC Cambonie G Marchand-Martin L Gournay V Durrmeyer X Durox M. Association between early screening for patent ductus arteriosus and in-hospital mortality among extremely preterm infants. JAMA. (2015) 313:2441–8. 10.1001/jama.2015.6734 26103028

[12] Ancel PY Goffinet F Group EW. EPIPAGE 2: a preterm birth cohort in France in 2011. BMC Pediatr. (2014) 14:97. 10.1186/1471-2431-14-97 24716860 PMC3991913

[13] Ancel PY Goffinet F Group E-W Kuhn P Langer B Matis J. Survival and morbidity of preterm children born at 22 through 34 weeks' gestation in France in 2011: results of the EPIPAGE-2 cohort study. JAMA Pediatr. (2015) 169:230–8. 10.1001/jamapediatrics.2014.3351 25621457

[14] Austin PC. A comparison of 12 algorithms for matching on the propensity score. Stat Med. (2014) 33:1057–69. 10.1002/sim.6004 24123228 PMC4285163

[15] Austin PC. Balance diagnostics for comparing the distribution of baseline covariates between treatment groups in propensity-score matched samples. Stat Med. (2009) 28:3083–107. 10.1002/sim.3697 19757444 PMC3472075

[16] Rubin DB Schenker N. Multiple imputation in health-care databases: an overview and some applications. Stat Med. (1991) 10:585–98. 10.1002/sim.4780100410 2057657

[17] Ego A Prunet C Lebreton E Blondel B Kaminski M Goffinet F. Courbes de croissance *in utero* ajustèes et non ajustèes adaptées à la population française. I - Méthodes de construction [Customized and non-customized French intrauterine growth curves. I - Methodology]. J Gynecol Obstet Biol Reprod. (2016) 45:155–64. 10.1016/j.jgyn.2015.08.009 26422365

[18] Schachinger S Stansfield RB Ensing G Schumacher R. The prevalence of and attitudes toward neonatal functional echocardiography use and training in the United States: a survey of neonatal intensive care unit medical directors. J Neonatal Perinatal Med. (2014) 7:125–30. 10.3233/NPM-1474013 25104118

[19] Corsini I Ficial B Fiocchi S Schena F Capolupo I Cerbo RM. Neonatologist performed echocardiography (NPE) in Italian neonatal intensive care units: a national survey. Ital J Pediatr. (2019) 45:131. 10.1186/s13052-019-0721-z 31640752 PMC6805655

[20] McNamara PJ Barker P Jain A Lai WW. Towards use of POCUS to evaluate hemodynamics in critically ill neonates: caution before adoption in this population. Crit Care. (2021) 25:92. 10.1186/s13054-020-03394-4 33658049 PMC7927214

[21] Subhedar NV Shaw NJ. Dopamine versus dobutamine for hypotensive preterm infants. Cochrane Database Syst Rev. (2003) 3:CD001242. 10.1002/14651858.CD001242 12917901

[22] Osborn DA Evans N. Early volume expansion for prevention of morbidity and mortality in very preterm infants. Cochrane Database Syst Rev. (2004) 2:CD002055. 10.1002/14651858.CD002055.pub2 15106166 PMC7025803

[23] Paradisis M Osborn DA. Adrenaline for prevention of morbidity and mortality in preterm infants with cardiovascular compromise. Cochrane Database Syst Rev. (2004) 1:CD003958. 10.1002/14651858.CD003958.pub2 14974048 PMC12159978

[24] Ibrahim H Sinha IP Subhedar NV. Corticosteroids for treating hypotension in preterm infants. Cochrane Database Syst Rev. (2011) (12):CD003662. 10.1002/14651858.CD003662.pub4 22161379 PMC7133776

[25] Dempsey EM Barrington KJ Marlow N O'Donnell CPF Miletin J Naulaers G. Hypotension in Preterm Infants (HIP) randomised trial. Arch Dis Child Fetal Neonatal Ed. (2021) 106:398–403. 10.1136/archdischild-2020-320241 33627329 PMC8237176

[26] Paradisis M Evans N Kluckow M Osborn D. Randomized trial of milrinone versus placebo for prevention of low systemic blood flow in very preterm infants. J Pediatr. (2009) 154:189–95. 10.1016/j.jpeds.2008.07.059 18822428

[27] Huang S Sanfilippo F Herpain A Balik M Chew M Clau-Terre F. Systematic review and literature appraisal on methodology of conducting and reporting critical-care echocardiography studies: a report from the European society of intensive care medicine PRICES expert panel. Ann Intensive Care. (2020) 10:49. 10.1186/s13613-020-00662-y 32335780 PMC7183522

[28] Stranak Z Semberova J Barrington K O'Donnell C Marlow N Naulaers G. International survey on diagnosis and management of hypotension in extremely preterm babies. Eur J Pediatr. (2014) 173:793–8. 10.1007/s00431-013-2251-9 24390060 PMC4032643

[29] Aldana-Aguirre JC Deshpande P Jain A Weisz DE. Physiology of low blood pressure during the first day after birth among extremely preterm neonates. J Pediatr. (2021). 10.1016/j.jpeds.2021.05.026 34019882

[30] Concato J Horwitz RI. Beyond randomised versus observational studies. Lancet. (2004) 363:1660–1. 10.1016/S0140-6736(04)16285-5 15158623

