# Supplementary material for: In-hospital Outcomes and Early Hemodynamic Management According to Echocardiography Use in Hypotensive Preterm Infants: A National Propensity-Matched Cohort Study
Source: Front Cardiovasc Med. 2022 Jul 14;9:852666. doi: 10.3389/fcvm.2022.852666 (PMC9329625; doi:10.3389/fcvm.2022.852666)
Supplement: Supplementary file 1 [file Data_Sheet_1.DOCX]

**In-hospital outcomes and early hemodynamic management according to echocardiography use in hypotensive preterm infants: a national propensity-matched cohort study – Supplementary Material**

**Methods:** Variables used for antihypotensive treatments and main reason declared by the attending physician for using such therapy

**Methods:** List of covariates included in the propensity score

**Methods:** Sensitivity analyses

**Results:** Supplementary Figure 1: Propensity score distribution in the no-NPE and NPE groups

**Results:** Supplementary Figure 2: Sensitivity analyses: Primary and secondary outcomes with inverse probability of treatment weighting (IPTW) and adjusted generalized estimation equation regression analysis (GEE)

**Results:** Supplementary Table 1. Subgroup analysis for primary outcomes among infants with minMAP ≤ or > GA-5 in the propensity score-matched cohort

**Results:** Supplementary Table 2. Subgroup analysis for primary outcomes among infants who did or did not receive any antihypotensive treatment in the first three days of life in the propensity score-matched cohort

**Variables used for antihypotensive treatments and main reason declared by the attending physician for using such therapy**

Antihypotensive treatments were categorized as volume expansion, inotropic drugs, and corticosteroids. All possible combinations of these categories were analyzed. The main reasons for administering them were categorized in the questionnaire as: isolated hypotension; hypotension associated with clinical signs of hypoperfusion; echocardiographic findings only; echocardiographic findings and clinical signs of hypoperfusion; other items suggesting hemodynamic compromise (e.g., laboratory results such as lactate or near infrared spectroscopy). Since NPE use for hemodynamic assessment could also include PDA assessment, PDA treatment, its nature, and date were also collected.

**List of covariates included in the propensity score**

The maternal and pregnancy variables were: maternal age (<25, 25-35, or > 35 years), antenatal corticosteroid administration, tocolysis use, antenatal magnesium sulphate administration, cause of prematurity categorized in four classes: preterm premature rupture of membranes, preterm labor, hypertensive disorders, and other causes (e.g., placental abruption, triplet or quadruplet births), mode of delivery (caesarean or vaginal), maternal anesthesia (general, epidural, or none), and multiple pregnancy. The neonatal characteristics at birth were gestational age, sex, small-for-gestational age (defined as a birth weight < 10th centile for gestational age and sex based on French intrauterine growth curves),(16) delayed cord clamping, 5-minute Apgar score <7, metabolic acidosis (defined as base deficit <-7 in the first 12 hours after birth), number of surfactant administrations (0, 1, or ≥ 2), intubation in the delivery room, continuous positive airway pressure attempted in the first 24 hours after birth, inhaled nitric oxide before day three, high frequency oscillatory ventilation before day eight, suspected early-onset sepsis, sedative or analgesic treatment before day three, systematic PDA echocardiographic screening before day three, severe hypotension (defined as minMAP ≤ GA – 5), inborn status, and the volume of activity of the unit where the infant was born, defined by the number of infants included in the EPIPAGE-2 study in the unit, divided into quartiles.

**Sensitivity analyses**

First, to assess the average treatment effect related to NPE use in the entire population (ATE), we used inverse probability of treatment weighting (IPWT) with the propensity score described above. In this analysis, observations were assigned weights proportional to the inverse of their probability of undergoing NPE, given their baseline covariates. Second, we compared outcomes between exposed and non-exposed infants with a GEE regression analysis to take into account a potential center effect, adjusted for gestational age (GA), birth weight < 10th centile, 5-minute Apgar score < 7, number of surfactant doses, attempted CPAP in the first 24 hours after birth, minMAP ≤ GA-5, and systematic PDA echocardiographic screening before day three. These two sensitivity analyses used multiple imputations as described in the main text.

**Supplementary Figure 1: Propensity score distribution in the no-NPE and NPE groups**


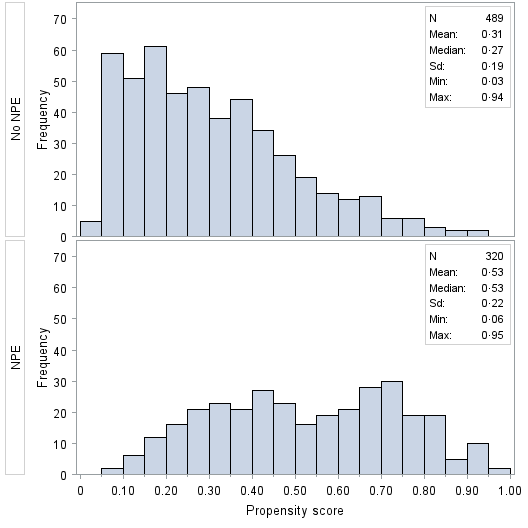


**Supplementary Figure 2. Sensitivity analyses: Primary and secondary outcomes with inverse probability of treatment weighting (IPTW) and adjusted generalized estimation equation regression analysis (GEE)**


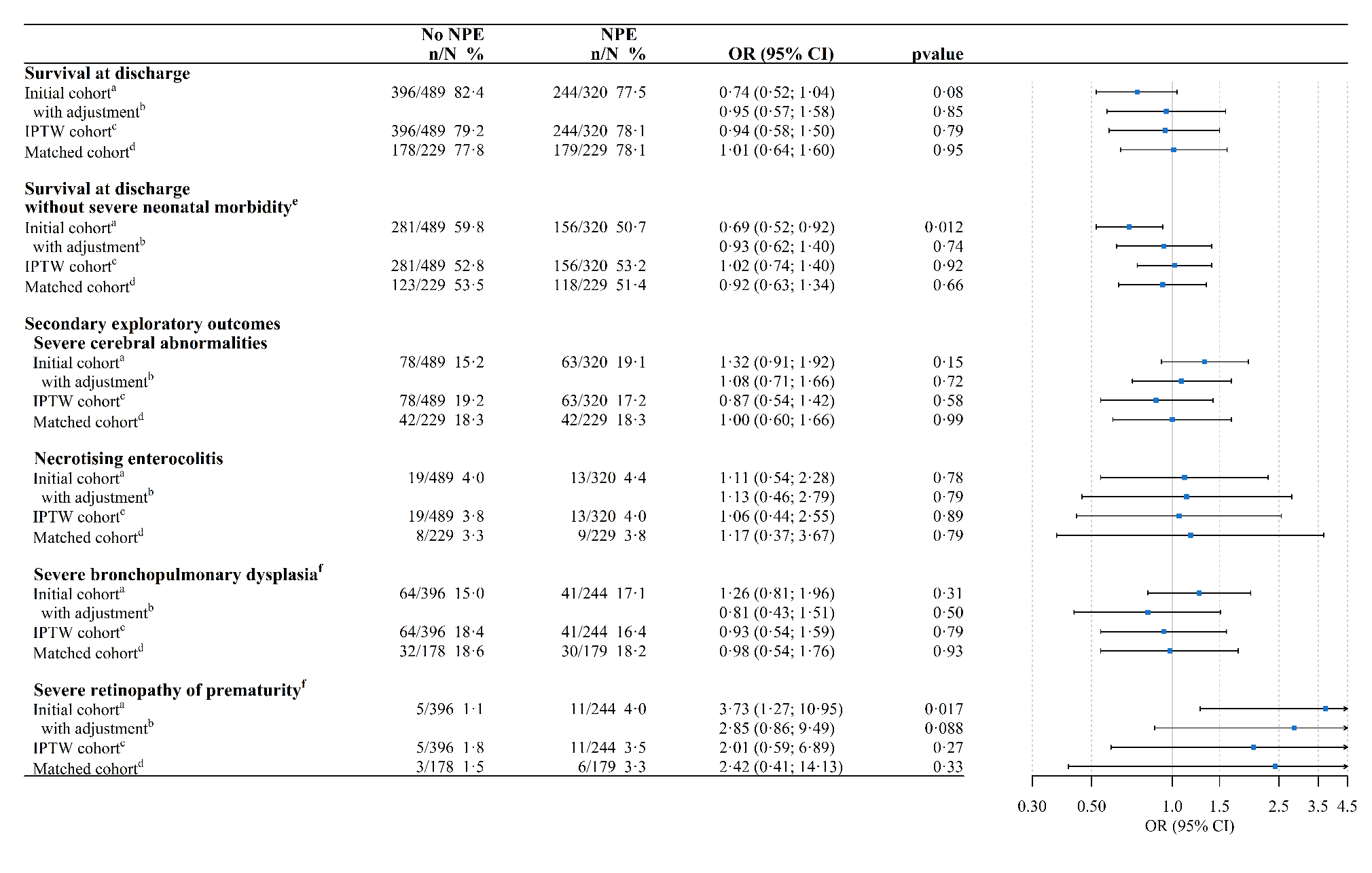


a Weighted to take differences in sampling process by gestational age into account. After multiple imputation. Mean numbers among the 50 imputed datasets.

b Generalized estimation equation regression analysis (GEE) to take into account a potential center effect, adjusted for gestational age (GA), birth weight < 10th centile, 5-min Apgar score < 7, number of doses of surfactant, attempted CPAP in the first 24 hours after birth, minMAP ≤ GA-5 and systematic DA echocardiographic screening before D3, after multiple imputation. Mean numbers among the 50 imputed datasets.

c Weighted by the inverse probability of treatment (IPWT). Generalized estimation equation regression analysis (GEE) to take a potential center effect into account.

d Matching by gestational age in weeks and propensity score after multiple imputation. Mean numbers among the 50 imputed datasets. Generalized estimation equation regression analysis (GEE) to take paired data into account.

e Severe morbidity was defined as any of: severe bronchopulmonary dysplasia, severe necrotizing enterocolitis, or severe retinopathy (stage 3 or treatment needed), or any of the following severe cerebral abnormalities on cranial ultrasonography: intraventricular hemorrhage with ventricular dilatation (Grade III IVH) or intraparenchymal hemorrhage, or cystic periventricular leukomalacia.

f Among survivors at discharge.

Abbreviations: IPTW, inverse probability of treatment weighting.

**Supplementary Table 1. Subgroup analysis for primary outcomes among infants with minMAP ≤ or > GA-5 in the propensity score-matched cohort^a^**

|  | **minMAP ≤ GA-5** | | | |  | **minMAP > GA-5** | | | |
| --- | --- | --- | --- | --- | --- | --- | --- | --- | --- |
|  | **No-NPE (n=76)** | **NPE (n=76)** | **OR (95%CI)** | **p-value** |  | **No-NPE (n=121)** | **NPE (n=121)** | **OR (95%CI)** | **p-value** |
|  | **n (%)** | **n (%)** |  |  |  | **n (%)** | **n (%)** |  |  |
| **Survival at discharge** | 58 (75·6) | 55 (72·8) | 0·86 (0·38; 1·96) | 0·72 |  | 97 (79·9) | 99 (82·0) | 1·15 (0·59; 2·21) | 0·69 |
| **Survival at discharge without severe neonatal morbidity^b^** | 33 (43·3) | 39 (51·3) | 1·38 (0·67; 2·84) | 0·38 |  | 70 (57·9) | 63 (52·5) | 0·81 (0·48; 1·35) | 0·41 |

a Matching by gestational age in weeks and propensity score after multiple imputation. Mean numbers among the 50 imputed datasets. Generalized estimation equation regression analysis (GEE) to take paired data into account. Analyses were performed with a new propensity score within each subgroup.

b Severe morbidity was defined as any of: severe bronchopulmonary dysplasia, severe necrotizing enterocolitis, or severe retinopathy (stage 3 or treatment needed), or any of the following severe cerebral abnormalities on cranial ultrasonography: intraventricular hemorrhage with ventricular dilatation (Grade III IVH) or intraparenchymal hemorrhage, or cystic periventricular leukomalacia.

Abbreviations: minMAP, minimum mean arterial blood pressure

**Supplementary Table 2. Subgroup analysis for primary outcomes among infants who did or did not receive any antihypotensive treatment in the first three days of life in the propensity score-matched cohort^a^**

|  | **Antihypotensive treatment before day 3 of life** | | | | |  | | **NO antihypotensive treatment before day 3 of life** | | | | |
| --- | --- | --- | --- | --- | --- | --- | --- | --- | --- | --- | --- | --- |
|  | **No-NPE (n=113)** | **NPE (n=113)** | **OR (95%CI)** | **p-value** |  | | **No-NPE (n=74)** | | **NPE (n=74)** | **OR (95%CI)** | **p-value** |  |
|  | **n (%)** | **n (%)** |  |  |  | | **n (%)** | | **n (%)** |  |  |  |
| **Survival at discharge** | 82 (72·5) | 82 (72·3) | 0·99 (0·52; 1·89) | 0·98 |  | | 59 (80·6) | | 63 (85·2) | 1·39 (0·54; 3·53) | 0·49 |  |
| **Survival at discharge without severe neonatal morbidity^b^** | 61 (53·8) | 56 (49·4) | 0·84 (0·48; 1·47) | 0·53 |  | | 41 (55·5) | | 39 (52·7) | 0·89 (0·44; 1·82) | 0·75 |  |

a Matching by gestational age in weeks and propensity score after multiple imputation. Mean numbers among the 50 imputed datasets. Generalized estimation equation regression analysis (GEE) to take paired data into account. Analyses were performed with a new propensity score within each subgroup.

b Severe morbidity was defined as any of: severe bronchopulmonary dysplasia, severe necrotizing enterocolitis, or severe retinopathy (stage 3 or treatment needed), or any of the following severe cerebral abnormalities on cranial ultrasonography: intraventricular hemorrhage with ventricular dilatation (Grade III IVH) or intraparenchymal hemorrhage, or cystic periventricular leukomalacia.
